# Paradigm shift: the primary function of the “Adiponectin Receptors” is to regulate cell membrane composition

**DOI:** 10.1186/s12944-021-01468-y

**Published:** 2021-04-30

**Authors:** Marc Pilon

**Affiliations:** Dept. Chemistry and Molecular Biology, Univ. Gothenburg, Box 462, S-405 30 Gothenburg, Sweden

**Keywords:** Adiponectin receptor, Membrane fluidity, Phospholipid, Desaturase, Ceramidase, *Caenorhabditis elegans*, Fatty acid, Lipid metabolism, LRIG, PAQR

## Abstract

The ADIPOR1 and ADIPOR2 proteins (ADIPORs) are generally considered as adiponectin receptors with anti-diabetic properties. However, studies on the yeast and *C. elegans* homologs of the mammalian ADIPORs, and of the ADIPORs themselves in various mammalian cell models, support an updated/different view. Based on findings in these experimental models, the ADIPORs are now emerging as evolutionarily conserved regulators of membrane homeostasis that do not require adiponectin to act as membrane fluidity sensors and regulate phospholipid composition. More specifically, membrane rigidification activates ADIPOR signaling to promote fatty acid desaturation and incorporation of polyunsaturated fatty acids into membrane phospholipids until fluidity is restored. The present review summarizes the evidence supporting this new view of the ADIPORs, and briefly examines physiological consequences.

## Background

The ADIPOR1 and ADIPOR2 proteins (ADIPORs) have been the subject of several high-profile articles suggesting that they are adiponectin receptors with anti-diabetic properties [1–5]. Separately, the yeast and *C. elegans* homologs of the mammalian ADIPORs have now been studied for over ten years and this is a brief account of what has been learned during that time.

## The ADIPORs are PAQR proteins

The ADIPORs are members of the PAQR (progestin and adipoQ receptors) protein family, named after two of its founding members [6]. There are eleven PAQR proteins in the human genome (PAQR1-11), and ADIPOR1 and ADIPOR2 correspond to PAQR1 and PAQR2, respectively. PAQR proteins have three important characteristics: (1) seven transmembrane domains; (2) an orientation inverse that of GPCRs (G protein-coupled receptors), i.e. with their N-terminus being cytoplasmic and the C-terminus extracellular; and (3) they are part of the larger CREST protein family of hydrolases [7]. ADIPOR1 and ADIPOR2 were initially identified as putative receptors for the primarily adipocyte-secreted protein adiponectin (other tissues can produce adiponectin but adipocytes are by far the main source [8]), which was done by screening a cDNA expression library to identify proteins that bound fluorescent-tagged bacterially expressed recombinant adiponectin [3]. Several publications have since reported adiponectin-dependent ADIPOR signaling or physiological roles [1, 2, 4, 5, 9, 10]. Cautiously however, one study suggest that the ADIPORs may not be adiponectin receptors [11], and adiponectin itself is now emerging as a lipid carrier protein, which would explain its high concentration in plasma [12]. The crystal structure of the ADIPORs has been solved and consists of a barrel-shaped conformation open towards the cytoplasm with a cavity capable of accommodating fatty acids (FAs), or FA-like substrates, and a zinc-coordination site that may either stabilize the structure and/or participate in a hydrolytic reaction, such as the proposed ceramidase activity first suggested by studies of the yeast homologs and consistent with observations with the mammalian proteins [5, 13, 14].

## Lessons from yeast

The yeast *Izh1, -2, -3* and *− 4* (implicated in zinc homeostasis) genes encode PAQR proteins similar to the ADIPORs. A 2004 study by Lyons et al. showed that the *Izh* genes regulate the levels of structural membrane sterols and, in this indirect way, may affect the permeability of certain ions, such as zinc [15]. Importantly, expression of *Izh* genes is regulated by an oleate response element (ORE) [16], and is inhibited by the presence of oleic acid (C18:1) or linoleic acid (C18:2), two unsaturated fatty acids (UFAs) that promote membrane fluidity when present in phospholipids [17]. Conversely, *Izh* gene expression is stimulated by the presence of saturated fatty acids (SFAs) that promote membrane rigidification when incorporated into phospholipids [15, 17]. In 2009, the Lyons group showed that IZH2 is a ceramidase, i.e. can hydrolyze ceramides to generate free FAs and sphingoid bases that act as second messengers [18]. The same group showed that the human ADIPOR1 and ADIPOR2 proteins can functionally mimic IZH proteins [19], and are functional as ceramidases when heterologously expressed in yeast cells [20]. The activity of the ADIPORs was enhanced by addition of adiponectin in the media during those experiments [20]; unfortunately, the source of adiponectin was not specified, which is an important point given that active recombinant adiponectin is difficult to produce and may contain biologically active impurities [21]. A separate study further strengthened the connection to membrane homeostasis by identifying genes mis-regulated in the *Izh2* mutant: down-regulated genes included several lipid metabolism genes such as *Ino1* (inositol-3-phosphate synthase), *Cho1* (phosphatidylserine synthase), *Tsc* (important for sphingosine synthesis) and *Erg28* (ergosterol biosynthesis); over-expressed genes included genes important for phosphate transport (*Pho84*, *Pho89* and *Pic2*) [22]. *Izh2* is also upregulated under condition of increased membrane rigidity in a yeast model with varying UFA content [23], and the *Izh2* and *Izh4* genes are continuously repressed under conditions of high UFAs, i.e. when membranes are not challenged by SFA-driven rigidification [24]. In summary, the yeast homologs of the ADIPORs regulate membrane composition (structural lipids), are up-regulated by membrane rigidification (e.g. excess SFAs), are inhibited by the presence of membrane-fluidizing UFAs, carry a ceramidase activity, signal via sphingoid bases and can be functionally replaced, at least partially, by the human ADIPORs.

## Lessons from ***C. elegans***

There are 5 PAQR proteins encoded by the *C. elegans* genome, two of which are clear ADIPOR homologs and are named PAQR-1 and PAQR-2 (there are no adiponectin homologs in *C. elegans*) [25]. Mutants lacking PAQR-1 have no obvious phenotypes, though they enhance the phenotypes of worms lacking PAQR-2, indicating redundancy [25, 26]. Worms lacking PAQR-2 have a deformed tail tip, impaired autophagy, reduced life span, brood size and locomotion, and are cold- and SFA-intolerant because both conditions promote membrane rigidification [25, 27–31]. Importantly, the primary defect in mutants lacking PAQR-2 is an excess of SFAs in their phospholipids accompanied by debilitating membrane rigidity, which we documented through lipidomics analysis and verified *in vivo* using fluorescence recovery after photobleaching (FRAP) [27, 28, 32–35]. A forward genetics screen led to the identification of the protein IGLR-2 as an obligate partner for PAQR-2 function: both proteins must be present in the same cell for systemic maintenance of membrane fluidity when worms are challenged with either cold temperatures or SFA-rich diets [28, 35]. IGLR-2 has a large extracellular N-terminal region containing one immunoglobulin domain and several leucine-rich repeats, one transmembrane alpha helix and a large cytoplasmic C-terminal domain [28]. IGLR-2 is loosely homologous to mammalian LRIG proteins, a family with nearly 40 members [36], though a functional IGLR-2 ortholog has not yet been identified. Bifluorescence complementation (BiFC) and fluorescence resonance energy transfer (FRET) studies have shown that IGLR-2 localizes to the plasma membrane where it interacts in a membrane rigidity-dependent manner with PAQR-2 [28, 37]. Additionally, structure-function studies indicate that the two proteins interact via their transmembrane domains and, more speculatively, that IGLR-2 may help displace the cytoplasmic PAQR-2 domain to facilitate access of substrates to the active site [26, 37]. Note also that IGLR-2 is not required for PAQR-1 function, suggesting that PAQR-1 either has a basal constitutive activity or is regulated via a separate mechanism [26].

**Fig. 1:**
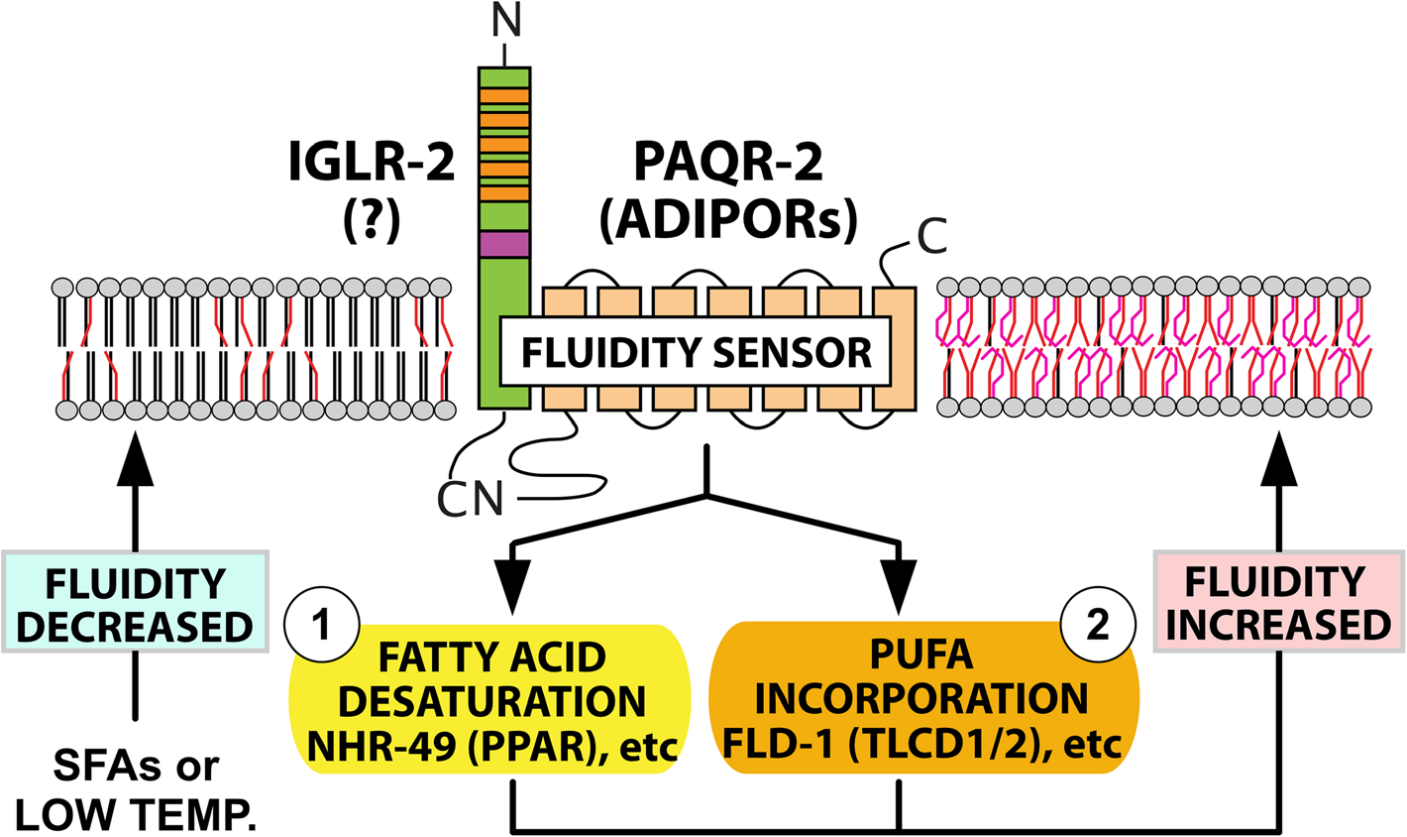
The role of PAQR-2 in membrane homeostasis. Conditions that promote membrane rigidification, such as SFA-rich diets or low temperature, stimulate the formation, hence activation, of the PAQR-2/IGLR-2 complex. While PAQR-2 is definitely an ADIPOR ortholog, no functional homolog of IGLR-2 has yet been identified in mammals. Genetic studies reveal that two downstream branches mediate the effects of the PAQR-2/IGLR-2 complex: (1) Branch 1 stimulates the transcription of fatty acid desaturase genes and can be replaced by gain-of-function mutations in NHR-49 (homologous to the mammalian PPARs), MDT-15 or SBP-1; and (2) Branch 2 stimulates the production/incorporation of PUFAs into phospholipids and can be replaced by loss-of-function mutations in FLD-1 (homologous to the mammalian TLCD1/2 proteins) or ACS-13. The ultimate output of PAQR-2 signaling is to increase the UFA content in phospholipids, which promotes fluidity

Remarkably, an unbiased genetic screen to identify mutants intolerant of dietary palmitic acid (a C16:0 SFA) showed that PAQR-2 and IGLR-2 are the only two *C. elegans* proteins specifically essential to prevent lethal membrane rigidification by SFA-rich diets [37]. Additionally, screens for secondary mutations that compensate for the lack of PAQR-2 revealed the existence of two independent downstream branches in the PAQR-2/IGLR-2 pathway: “Branch 1” acts by upregulating the expression of desaturases, and its function can be replaced by gain-of-function alleles of NHR-49 (a homolog of the mammalian PPARs), MDT-15 (a homolog of the mammalian mediator subunit MED15) or SBP-1 (a homolog of the mammalian SREBPs)[29]; “Branch 2” acts by promoting PUFA production and/or their incorporation into phospholipids, and its function can be replaced by loss-of-function mutations in FLD-1 (a homolog of the mammalian TLCD1 and TLCD2 proteins) or ACS-13 (a homolog of the mammalian ACSL1)[32, 33]. Only by simultaneously providing mutations that replace both branches can one achieve complete suppression of all phenotypes found in worms lacking PAQR-2 [32, 33](Fig. 1). In summary, work in *C. elegans* showed that the essential function of PAQR-2 is to respond to membrane rigidification by low temperature or SFA-rich diets by promoting a compensatory increase in phospholipids that contain UFAs.

## Translation to mammalian cells

The findings concerning *C. elegans* PAQR-2 have been extended to the ADIPORs in mammalian cells using siRNA and CRISPR/Cas9 to silence or knock out target genes, RNA sequencing to monitor the transcriptome, lipidomics to monitor the FA composition of phospholipids, and three different methods to measure membrane fluidity, namely FRAP, Laurdan dye staining and atomic force microscopy. We found that ADIPOR1 and/or ADIPOR2 depletion causes excess SFAs in phospholipids accompanied by membrane rigidification in all cell lines studied (embryonic kidney cells-derived HEK293, hepatocyte-derived HepG2, astrocyte-like 1321N1) and in primary human umbilical vein endothelial cells (HUVECs), with the most potent effects occurring with dual silencing of ADIPOR1 and ADIPOR2 [27, 32, 33, 38, 39]. qPCR and an exhaustive RNAseq analysis also showed that ADIPOR2 is required for a normal transcriptional response to SFA challenges: thousands of genes become mis-regulated in ADIPOR2-deficient cells challenged with 200 µM palmitate [39]. Among the most significantly downregulated genes in ADIPOR2-KO cells are the desaturases SCD, FADS1 and FADS2 while genes of the UPR (unfolded protein response) are among the most upregulated genes, which is likely a secondary consequence of the membrane homeostasis failure [39]. Not surprisingly, the ADIPOR-deficient cells exhibit mitochondrial respiration defects, poor viability and abnormal morphology when challenged with SFAs [39]. Most phenotypes found in cells lacking one or both ADIPORs can be abrogated by supplementing the cultures with membrane-fluidizing FAs such as oleic acid (a C18:1 UFA), eicosapentaenoic acid (a C20:5 PUFA) or docosahexaenoic acid (a C22:6 PUFA), which indicates that membrane rigidification is the primary defect in these cells [33, 38, 39]. Importantly, adiponectin is not required for the membrane homeostasis function of ADIPOR1 and ADIPOR2 in the cultured cell types tested, and the addition of a commercially purchased recombinant adiponectin produced from a mammalian expression system had no effect on the membrane fluidity of HEK293 cells [38]. This is somewhat confusing given the many studies documenting ADIPOR-dependent in vivo effects of adiponectin, including its effect on ceramide levels [5, 10]. Unfortunately, no comprehensive analysis of phospholipid composition has been reported for ADIPOR1/2 or adiponectin mutant mice, though it is known that ADIPOR1 mutations cause a severe depletion of long-chain PUFAs in the phosphatidylcholines of the retina accompanied by impaired vision both in mice and humans [40–43]. In terms of downstream events, it is likely that the two pathway branches mediating the effects of *C. elegans* PAQR-2/IGLR-2 are conserved in the human ADIPORs since they too regulate desaturase gene expression (“Branch 1”) [38], and their activity can at least partially be replaced by silencing TLCD1/TLCD2 or ACSL1, the homologs of *C. elegans* FLD-1 and ACS-13, respectively (“Branch 2”) [32, 33].

Recently, genome-wide CRISPR/Cas9 screens by the groups of Kivanc Birsoy (Rockefeller University) and Vamsi Mootha (Harvard Medical School) have provided further incontrovertible evidence that the ADIPORs are crucial for membrane homeostasis. In particular, ADIPOR2 ranked 25th out of ~ 3 000 metabolism genes tested for their ability to prevent palmitate toxicity in T-cell-derived Jurkat cells [44], and ranked 4th out of ~ 20 000 genes tested for their ability to suppress membrane rigidification in K562 cells challenged with hypoxia (a condition that inhibits oxygen-dependent desaturase reactions)[45]. Adiponectin itself was not picked by either screen, which may be due to the fact that it does not regulate ADIPOR2 or because it belongs to a family of secreted protein that may have partially redundant functions [46–49]. In conclusion, the results of ours and other’s studies are unambiguous: the function of ADIPOR1 and ADIPOR2 is conserved with that of *C. elegans* PAQR-2, and regulation of membrane homeostasis in most cell types is likely the ancestral function of these proteins, possibly extending as far back as the common ancestor between yeasts and animals. It is not known at present if there is a functional homolog to IGLR-2 in mammalian cells. Speculatively, functionally equivalent mechanisms (i.e. fluidity-dependent interactions) could involve other types of documented protein-protein interactions (e.g. APPL1; [50]) or homo/heterodimerization of the ADIPORs themselves [51, 52]. Also, future research will elucidate the precise nature of the two “branches” downstream of the ADIPORs, which likely involves their ceramidase activity.

## An updated view of the ADIPORs

So far, ADIPOR1 and ADIPOR2 have been primarily considered as adiponectin receptors that act via PPARα, AMPK, and/or ceramide depletion to improve insulin function and protect from the metabolic syndrome [3, 5, 9, 10, 53, 54]. The literature just now summarized suggests a different view: the ADIPORs are evolutionarily conserved regulators of membrane homeostasis that do not require adiponectin to act as membrane fluidity sensors and regulate phospholipid composition. More specifically, membrane rigidification activates ADIPOR signaling to promote fatty acid desaturation and incorporation of PUFAs into membrane phospholipids. In this view, impaired ADIPOR function primarily leads to increased intracellular SFA levels and membrane rigidity and, secondarily, to a panoply of side effects including elevated ceramide levels (a likely consequence of increased intracellular palmitate [5, 10, 55–58]), altered signaling by numerous receptors and increased susceptibility to several diseases.

## Relevance for human health

Membrane homeostasis is essential for most cellular processes, including membrane trafficking and fusion, organelle function, cytokinesis, etc. In particular, many receptors, transporters and channels require specific lipid interactions for their activity [59]. For example, SFAs induce c-SRC clustering, leading to the activation of JNK that in turns inhibit the insulin pathway and contributes to insulin resistance [60, 61]. Conversely, membrane fluidity increases insulin receptor signaling [62]. TRPV channels, which sense and regulate blood pressure in response to shear stress [63], are sensitive to membrane composition, requiring PUFA-containing phospholipids for their activation [64–66]. On the other hand, SFAs reduce membrane fluidity and promote the accumulation of TLR4 receptors in specialized membrane domains called lipid rafts, leading to homodimerization and proinflammatory signaling [67, 68]. Cell membrane tension and membrane fluidity also affect conformational dynamics of GPCRs and their ability to sense shear stress in endothelial cells [69]. And as a last example, phase separation of signaling molecules, which is influenced by membrane fluidity, promotes T cell receptor signal transduction [70]. This, merely the tip of a large iceberg, should suffice to convince any reader that membrane composition/property homeostasis is an important parameter in the regulation of vital signaling pathways. There are also numerous conditions where membrane fluidity defects are likely implicated: retinitis pigmentosa [41–43, 71], male sterility (in mice) [72–74], diabetes [75–82], X-linked adrenoleukodystrophy [83, 84], Gaucher disease [85], glycogen storage diseases [86], hypertension [87–92], thromboembolic disorders [93], cancer [94–103], Niemann-Pick disease type C [104], Parkinson’s disease [105–108], Alzheimer’s disease [109–113], Huntington’s disease [114, 115], Batten disease [116], polycystic kidney disease [117], inflammation [67, 68, 118, 119] and aging [120–124]. Note that the ADIPORs are ubiquitously expressed and that their roles in membrane homeostasis are cell non-autonomous due to lipid exchange among cells/tissues [35], which may complicate therapeutic approaches targeting specific organs. Nevertheless, pharmacological modulation of the ADIPOR pathway, possibly using agonists such as AdipoRon [125, 126], could have wide-ranging medical usefulness, and continued research to further elucidate the fluidity-sensing mechanisms and the pathways downstream of the ADIPORs will contribute to this development.

## Study strengths and limitations

A strength of this review is that it emphasizes recent work on the cell and molecular function of the ADIPORs with a focus on model systems such as yeast, the nematode *C. elegans* and cultured mammalian cells. Conversely, the review does not cover in details the much more complex in vivo roles where the physiology of the whole organism makes it difficult to deduce specific molecular functions.

## Conclusion, application and future perspective

The primary and evolutionarily conserved function of the ADIPORs is to regulate cell membrane composition to maintain its fluidity; other effects of the ADIPORs are likely secondary to this primary function. In the future, it will be interesting to elucidate the precise fluidity-sensing mechanism and the precise nature of the molecular events downstream of the ADIPORs by which they regulate the fatty acid composition of phospholipids.

## Data Availability

Not applicable.

## Abbreviations

ADIPOR: Adiponectin receptor
BiFC: Bifluorescence complementation
FA: Fatty acid
FRAP: Fluorescence recovery after photobleaching
FRET: Fluorescence resonance energy transfer
PAQR: Progestin and adipoQ receptor
SFA: Saturated fatty acid
UFA: Unsaturated fatty acid
